# Preoperative incidence and risk factors of deep vein thrombosis in patients with an isolated patellar fracture

**DOI:** 10.1186/s12891-022-05163-6

**Published:** 2022-03-03

**Authors:** Weijie Yang, Haicheng Wang, Qun Wei, Kai Ding, Yuxuan Jia, Chao Li, Yanbin Zhu, Wei Chen

**Affiliations:** 1 Trauma Emergency Center, Key Laboratory of Biomechanics of Hebei Province, the Third Hospital of Hebei Medical University, No. 139 Ziqiang Road, Qiaoxi District, Shijiazhuang, 050051 Hebei PR China; 2 Department of Hospital Infection Control, Department of Public Health, Hebei General Hospital, Shijiazhuang, 050051 Hebei PR China; 3 Department of Clinical Medicine, School of Basic Medicine, Hebei Medical University, Shijiazhuang, Hebei PR China

**Keywords:** Patellar fracture, DVT, Incidence, Risk factors

## Abstract

**Purpose:**

This study aimed to investigate the incidence, location, and related factors of preoperative deep venous thrombosis (DVT) in patients with isolated patellar fractures.

**Methods:**

Patients with an isolated patellar fracture, admitted between January 2013 and December 2019 at our institution, were retrospectively analyzed. Upon admission, patients underwent routine Doppler ultrasound scanning (DUS) of the bilateral lower extremities to detect DVT; those with DVT were assigned to the case group and those without DVT to the control group. Patients in both groups did not perform preoperative off-bed weight-bearing exercises. Data on demographics, comorbidities, and laboratory test results upon admission were extracted. Variables were evaluated between the two groups using univariate analyses, and independent risk factors associated with DVT were identified by logistic regression analysis.

**Results:**

During the study period, 827 patients were included, of whom 5.8% (48/827) were found to have preoperative DVT. In DVT patients, 85.4%(41/48) were injured, 8.3%(4/48) were not injured, and 6.3%(3/48) were lower limbs. Multivariate analysis showed that male (male vs. female, odds ratio, OR = 2.25), delayed from injury to DUS (in each day, OR = 1.29), and elevated plasma D-dimer level (> 0.5 µg/mL, OR = 2.47) were independent risk factors associated with DVT.

**Conclusions:**

Despite the low prevalence of DVT after an isolated patellar fracture, this study underscores the importance of identifying those with a high risk of DVT, especially those with multiple identifiable factors, and encourage the early targeted use of anti-thromboembolic agents to reduce DVT occurrence.

## Introduction

DVT of the lower extremities is one of the common complications in hospitalized patients, and it often leads to the occurrence of pulmonary embolism, especially in patients with traumatic fractures [1, 2]. Hypercoagulability of blood, damage to the inner wall of blood vessels, and slowing of blood flow velocity are the physiological basis for the development of DVT [3]. These factors are often present in patients following a fracture. For example, the hypercoagulability of blood caused by the inflammatory reaction after fracture [4], and the slowing of blood flow caused by fixed restriction after fracture can promote the occurrence of lower limb DVT after a fracture [5]. Therefore, early prevention and diagnosis of preoperative thrombosis are particularly important for shortening the fracture healing time and restoring the bone and joint function.

Based on thromboprophylaxis intervention, the incidence of lower extremity DVT in patients with fractures around the knee has been reported to be between 16.3% to 35% [6, 7], of which 6.6% died of a pulmonary embolism [8]. Patients with fractures around the knee often require surgical treatment to restore knee function. Preoperative DVT seriously affects the timing of surgery and prolongs the treatment period. The patella is an essential structure during knee extension, and the incidence of patellar fractures ranges from 0.13% to 0.61% [9, 10], accounting for 0.5% to 1.5% of the total incidence of fractures in adults [11, 12]. At present, studies on fractures around the knee are mostly designed for femur, tibia, or knee fractures. To this date, only two studies have studied the relationship between patellar fractures and DVT; they found that the incidence of perioperative DVT in patellar fractures was 0.3% to 9% [2, 13]. However, these studies did not differentiate between preoperative and postoperative the incidence of DVT. In addition, reports on risk factors for DVT were inconsistent across studies. For example, Zhang et al. [2] found that preoperative time and plasma d-dimer were independent risk factors for DVT. While Li et al. [13] found that age > 50 years, joint replacement, and operation duration exceeding 3 h were independent risk factors. However, only risk factors for DVT in patients with fractures of the entire lower limb were considered in these studies. Moreover, their studies have been inconsistent regarding risk factors for DVT after patellar fracture.

The purpose of our study is to retrospectively analyze the incidence, location, and risk factors of preoperative bilateral lower extremity DVT diagnosed by DUS of patients with isolated patella fracture admitted to our hospital.

## Materials and methods

### Patients

This study retrospectively collected information on patients with patellar fracture treated in our hospital from January 2013 to December 2019. This study was approved by the Ethics Committee of the Third Hospital of Hebei Medical University. The inclusion criteria were age > 18 years with an isolated patellar fracture requiring surgical treatment. The exclusion criteria were bilateral patellar fractures, multiple fractures, open fractures, old fractures and pathological fractures, use of blood circulation pumps, autoimmune diseases, anticoagulant use within 3 months of admission, and incomplete medical records.

After admission, all patients received routine basic prophylaxis (e.g. lower limb elevation and deep breath) and chemoprophylaxis (subcutaneous injection of low molecular weight heparin, 2500-4100 IU, once daily). All patients routinely received Intermittent Pneumatic Compression Devices(IPCD)to prevent thrombosis [14, 15]. For patients allergic to LMWH, oral rivaroxaban was administered at a dosage of 10 mg once daily. Owing to the simplicity of these prophylactic measures, patients had good compliance overall.

As per our policy, all patients were not allowed to perform any weight-bearing exercises during the preoperative period and were only allowed to perform ankle pump exercises and hook-toe exercises on the bed [16]. As from the first postoperative day, patients were encouraged to do equal-length exercise and straight leg tension exercises; and depending on the tolerance, non- or partial-weight-bearing mobilization with the help of crutches was performed. At 6–8 weeks, the brace was removed after radiograph findings show no evidence of bone union. Resistance exercise was started, and complete weight-bearing mobilization was allowed.

### Data collection

The data covered demographics, chronic comorbidities, and laboratory biomarkers. These include age, gender, body mass index (BMI), smoking, hypertension, diabetes, cerebrovascular disease, chronic heart disease, lung disease, any surgery history, time from injury to Doppler Ultrasound (DUS) examination, the American Society of Anesthesiologist (ASA) score; Laboratory tests included measurements of levels of total protein (TP), albumin (ALB), alanine transaminase (ALT), aspartate aminotransferase (AST), alkaline phosphatase (ALP), high-sensitivity C-reactive protein (HCRP), creatine kinase (CK), lactic dehydrogenases (LDH), total cholesterol (TC), triglycerides (TG), glucose (GLU), D-dimer, fibrinogen (FIB), fibrinogen degradation product (FDP), and routine blood examinations included: white blood cells (WBC), neutrophils (NEU), lymphocytes (LYM), red blood cell (RBC), hemoglobin (HGB) level, hematocrit (HCT), platelets (PLT), prothrombin time (PT), prothrombin activity (PTA), international normalized ratio (INR), activated partial thromboplastin time (APTT), and thrombin time (TT).

### Diagnosis criteria of thrombosis

After admission and before surgery, the patients were tested for DUS every 3 days, and if the surgery date was less than 3 days, DUS was performed a day before surgery. The "Guidelines for the Diagnosis and Treatment of Deep Vein Thrombosis (2016 3rd Edition)" issued by the Chinese Medical Association was used to diagnose and treat DVTs. Positive diagnostic criteria for DVT included (a) loss or incompressibility of the vein, (b) lumen obstruction or filling defects, (c) lack of respiratory variability in the vein segments above the knee, and (d) insufficient increase in blood flow during compression of the leg and foot. The common femoral vein, femoral vein, deep femoral vein, popliteal vein, posterior tibial vein, anterior tibial vein, and peroneal vein were assessed.

According to the thrombotic test criteria, the medical sonographer examined and reported the findings of the femoral vein trunk and the femoral deep, superficial, popliteal, tibial, and peroneal veins of both lower extremities. For patients with deep vein thrombosis, we only perform temporary fracture fixation after the thrombosis is first found, and then transfer the fracture to vascular surgery for diagnosis and treatment by professional vascular surgeons. After the condition is stable, orthopedic and vascular surgeons will evaluate the timing of surgery. Proximal DVT was defined as thrombi in the popliteal vein and above. The DVTs below the popliteal vein were considered as distal DVT. The clinical significance of thrombi in the intermuscular vein, small saphenous vein, and the great saphenous vein was relatively small; therefore, they were excluded from this study [17].

### Statistical analysis

SPSS 25.0 software (IBM, Armonk, New York, USA) was used for statistical analysis. The measurement data were first explored using the Shapiro–Wilk test for their distribution status (normal or non-normal). Normal distribution data were expressed as mean ± standard deviation (Sd), and an independent sample t-test was used to compare the differences between groups. The Mann–Whitney U test was used for non-normally distributed data. Categorical variables were assessed using the chi-square or Fisher's exact tests. *P* values < 0.10 in the univariate analyses were further analyzed by multivariate logistic regression. *P* values < 0.05 were considered statistically significant for all analyses.

## Results

**Fig. 1:**
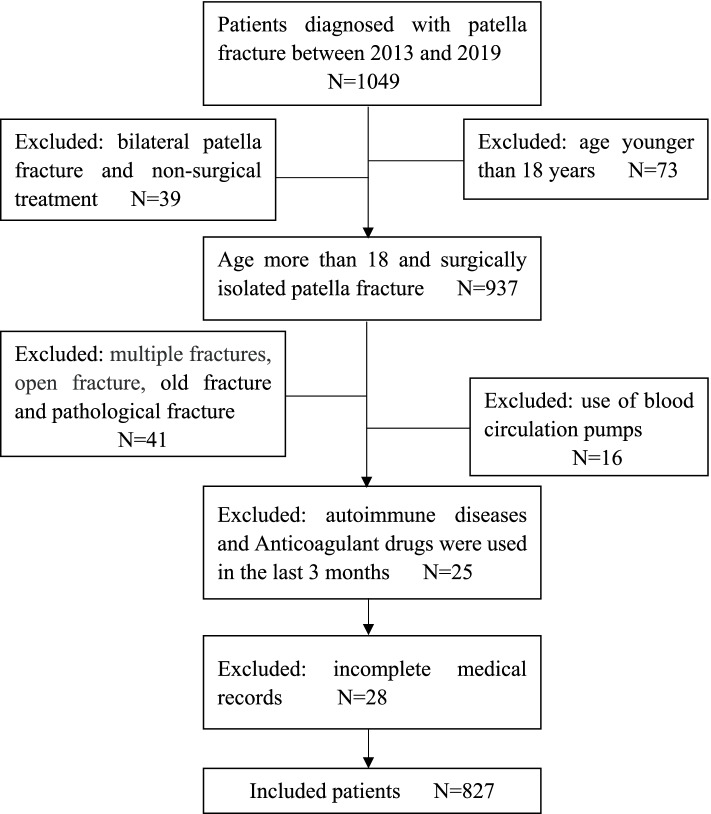
Flow chart of patient inclusion and exclusion in this study

A total of 1,049 patients with patellar fractures were admitted during the study period. Among them, 73 patients were excluded because they were younger than 18 years; 39 due to bilateral patellar fracture and non-surgical treatment; 41 due to multiple fractures, open fractures, old fractures, and pathological fractures; 16 due to use of blood circulation pumps, 25 due to autoimmune diseases, and use of anticoagulant drugs during the past 3 months and 28 due to incomplete medical records (Fig. 1).

A total of 827 patients with patellar fractures were included in this study. Of these, 63.0% (521/827) were males, and 37% (306/827) were females, with an average age of 51.9 years (Sd, 14.9; range, 18–92 years), and 20.1% (166/827) were aged 65 years or above. The average BMI was 24.6 (Sd, 3.4; range 17.3–46.7). The mean days from admission to operation in the non-DVT and DVT groups were 4.2 ± 2.8 days and 8.7 ± 4.6 days, respectively (Table 1).

**Table 1:** Univariate analyses of risk factors associated with preoperative DVT following Patellar fracture

Variables	NO. (%) without DVT (*n* = 779)	NO. (%) with DVT (*n* = 48)	*P* value
Gender			0.017
Man	483 (62.0)	38 (79.2)	
Woman	296 (38.0)	10 (20.8)	
Age (years)	51.6 ± 14.8	56.9 ± 15.4	0.016
Smoking	88 (11.3)	9 (18.8)	0.119
BMI, kg/m^2^	24.7 ± 3.3	24.2 ± 3.0	0.407
< 18	182 (23.3)	17 (37.4)	
18–23.9	268 (34.4)	18 (37.5)	
24.0–27.9	256 (32.9)	8 (16.7)	
≥ 28	73 (9.4)	5 (10.4)	
Hypertension	175 (22.5)	11 (22.9)	0.942
Diabetes	94 (12.1)	5 (10.4)	0.733
Cerebrovascular disease	58 (7.4)	5 (10.4)	0.451
Chronic heart disease	182 (23.4)	15 (31.3)	0.213
Lung disease	5 (0.6)	1 (2.1)	0.253
Surgery history	147 (18.9)	7 (14.6)	0.459
Mean days from admission to operation (days)	4.2 ± 2.8	8.7 ± 4.6	0.001
Time from injury to DUS (days)	3.1 ± 2.7	7.6 ± 4.5	0.001
Time from injury to anticoagulation (days)	1.1 ± 0.7	1.3 ± 0.5	0.351
ASA			0.488
I-II	691 (88.7)	41 (85.4)	
III-IV	88 (11.3)	7 (14.6)	
TP (< 60 g/L)	70 (9.0)	8 (16.7)	0.077
ALB (< 35 g/L)	59 (7.6)	9 (18.8)	0.006
ALT (> upper limit)	61 (7.8)	8 (16.7)	0.032
AST (> upper limit)	31(4.0)	3 (6.3)	0.442
ALP (> upper limit)	8 (1.0)	2 (4.2)	0.053
HCRP (> 8 mg/L)	361 (46.3)	28 (58.3)	0.106
CK (> upper limit)	63 (8.1)	5 (10.4)	0.569
LDH (> 250 U/L)	61 (7.8)	7 (14.6)	0.098
TC (> 5.8 mmol/L)	124 (15.9)	5 (10.4)	0.308
TG (> 1.7 mmol/L)	150 (19.3)	9 (18.8)	0.931
GLU (> 6.1 mmol/L)	255 (32.7)	18 (37.5)	0.496
WBC (> 10*10^9^/L)	182 (23.4)	17 (35.4)	0.058
NEUT (> 6.3*10^9^ /L)	314 (40.3)	27 (56.3)	0.029
LYMT (< 1.8*10^9^ /L)	167(21.4)	13 (27.1)	0.358
RBC (< lower limit)	195 (25.0)	14 (29.2)	0.522
HGB (< lower limit)	142 (18.2)	11 (22.9)	0.417
HCT (< lower limit)	341 (43.8)	23 (47.9)	0.575
PLT (> 300*10^9^ /L)	24 (3.1)	4 (8.3)	0.051
PT (> 12.5 s)	9 (14.0)	1 (14.6)	0.568
PTA (< 80%)	46 (5.9)	3 (6.3)	0.922
INR (> 1.4)	25 (3.2)	1 (2.1)	0.664
APTT (< 28 s)	219 (28.1)	18 (37.5)	0.163
TT (> 17 s)	81 (10.4)	3 (6.3)	0.356
FIB (> 4 g/L)	118 (15.1)	11 (22.9)	0.150
FDP (> 5 mg/L)	129 (16.6)	13 (27.1)	0.061
D-dimer (> 0.5 ug/mL)	205 (26.3)	33 (68.8)	0.001

*Notes*: *ALT* Alanine transaminase, reference range: female, 7–40 U/L; male, 9–50 U/L; *AST* Aspartate transaminase, reference range: female, 13–35 U/L; male, 15–40 U/L; *ALP* Alkaline phosphatase, reference range: female, 35–100 U/L; male, 45–125 U/L; *CK* Creatine kinase, reference range: female, 40–200 U/L; male, 50–310 U/L; *CREA* creatinine, reference range: female, 41–73 umol/L; male, 57–111 umol/L; *UA* Uric acid; reference range: female, 155–357 umol/L; male, 208–248 umol/L; *RBC* Red blood cell, reference range: Female, 3.5–5.0*10^12^/L; male, 4.0–5.5*10^12^/L; *HGB* Hemoglobin, reference range: Female, 110-150 g/L; male, 120-160 g/L; *HCT* Hematocrit, reference range: Female, 35–45%; male, 40–50%

Among the DVT patients, 5.8% (48/827) were found to have preoperative DVT, with 79.2% (38/48) in males and 20.8% (10/48) in females. Their average age was 57.0 years (Sd, 15.5, range 28–90 years). The average BMI was (24.2 ± 3.02); range (18.7–30.1). In addition, 25% (12/48) of patients were 65 years or older. From injury to DVT diagnosis, the average time was (7.6 ± 4.5); range (1–24 days). All DVTs were asymptomatic.

Among the DVT patients, 22 had left-sided fractures, and 26 had right-sided fractures. Of the 22 patients with left-sided fracture, 21 had thrombus in the injured extremity, and 1 had thrombus in the bilateral extremity. Among the 26 patients with right-sided fractures, the thrombus was found in the injured extremity, uninjured extremity, and bilateral extremity in 20, 4, and 2 patients, respectively (Table 2). Among the patients with DVT, there were 9 (18.8%) proximal DVT and 39 (81.2%) distal DVT, respectively (Table 3). The distribution of thrombus in blood vessels was as follows: 1 in the femoral superficial vein, 7 in the popliteal veins, 20 in the peroneal veins, and 20 in the posterior tibial veins in patients with left-sided fractures; and 1 in the femoral superficial vein, 2 in the popliteal veins, 15 in the peroneal veins, and 9 in the posterior tibial veins in patients with right-sided fractures. None of the patients developed preoperative pulmonary embolism.

**Table 2:** Distribution of thrombus in lower extremities

Fracture sides	Thrombus			
	Injured extremity	Uninjured extremity	Bilateral	Total
Left fracture	21 (95.4)	0 (0.0)	1 (4.6)	22 (100)
Right fracture	20 (76.9)	4 (15.4)	2 (7.7)	26 (100)
Total	41 (85.4)	4 (8.3)	3 (6.3)	48 (100)

**Table 3:** The distribution of thrombus in blood vessels

Fracture sides	Proximal DVT	Distal DVT	Total
Left fracture	2 (9.1)	20 (90.9)	22 (100)
Right fracture	7 (26.9)	19 (73.1)	26 (100)
Total	9 (18.8)	39 (81.2)	48 (100)

Comparison of the variables between the DVT and non-DVT groups showed statistically significant differences in sex, age, time from injury to DUS, ALB lower limit, ALT upper limit, NEUT upper limit, and D-dimer upper limit (*P* < 0.05, Table 1).

In the multivariate logistic regression model analysis, age (each increase 1 year), (OR = 1.02), delay from injury to DUS (in each day, OR = 1.33), and elevated plasma D-dimer level (> 0.5 µg/mL, OR = 2.47) were identified as independent risk factors for preoperative DVT (Table 4).

**Table 4:** Multivariate analyses of risk factors associated with preoperative DVT after patellar fracture

Variable	OR and 95%CI	*P* value
Gender (male us female)	2.25 (1.04–4.90)	0.040
Time from injury to DUS (in each day delay)	1.29 (1.20–1.39)	0.001
D-dimer level (> 0.5 ug/ml)	2.28 (1.61–3.22)	0.001

## Discussion

DVT is a common complication in patients with fractures, potentially affecting their prognosis. However, there are many uncertainties about the risk factors associated with thrombus formation. It is therefore essential to understand the significance of preoperative DVT in patients with isolated patellar fractures. In this study, we found that the preoperative incidence of DVT after patellar fracture was 5.8% (48/827), and 85.4% (41/48) of those occurred in the injured extremity. We also found that the associated risk factors were age, time from injury to DUS, and plasma D-dimer level. DVT is a common problem in patients with fractures and has been widely discussed in clinical practice [18]. We found a 5.8% (48/827) preoperative incidence of DVT after an isolated patellar fracture. However, Wang et al. [19] reported an incidence of DVT in 15.3% of 59 patellar fractures. Li et al. [13] also reported an overall 8.2% rate in lower extremity fractures, with 9% of DVT in 177 knee fractures. The use of DUS, compared to the use of computed tomography (CT) or magnetic resonance imaging (MRI) venography for the detection of DVTs, may partly explain the relatively large gap in incidence rates. We also found that DVTs occurred not only in the injured extremity but also in the non-injured extremity, which is consistent with the findings of Wang et al. [20] This is due to the hypercoagulable state of blood after fracture and the patient's long-term lack of exercise in bed, which leads to not only the affected limb prone to thrombosis, but also the healthy limb prone to thrombosis. This suggests the equal importance of screening for DVT in both the non-injured and injured extremities.

Among patients with a patellar fracture in this study, the risk of DVT in men was 2.25 times that of women, and the average age was 56.9 years, indicating that middle-aged and older men were risk factors for DVT following a patellar fracture. Similarly, men were also found to be a risk factor for perioperative DVT in two previous studies on tibial fractures and ankle fractures [21, 22]. Estrogen is a risk factor for blood clots in younger women, while lower hormone levels in older women reduced DVT risk in women [23]. In line with this view, scholars also found that postmenopausal women are less likely to develop DVT [24]. Therefore, estrogen levels in the body may be responsible for the discrepancies between gender and the risk of DVT development found in our study.

Our results showed that the preoperative duration was significantly longer in the DVT group than in the non-DVT group, with a 33% increased risk of preoperative DVT for each day of delay from the injury to DUS. Consistently, Zuo et al. [25] found that the duration before admission to the hospital in the DVT group was longer than in the non-DVT group after intertrochanteric fractures, and the risk of DVT was increased by 37% each day of delay to admission. The hypercoagulable state after trauma is the pathophysiological basis of DVT. In particular, the blood coagulation dynamic value was the highest in the first 24 h after the trauma and remained hypercoagulable during the first 4 days [26]. Similar conclusions were reached in the study conducted by Decker et al. [27] Therefore, we recommend routine DVT screening for all fracture patients after admission and surgery within 24–48 h after injury, which should provide the feasibility of reducing perioperative adverse events [28]. In addition, more attention should be paid to patients with delayed admission of more than 3 days to avoid missed diagnosis of DVT. Fractures are often accompanied by crush injuries, which can cause bleeding to compress veins, resulting in hemodynamic disruption, as well as damage to the veins themselves, particularly the endothelial cells. These can lead to blood clots. We suggest that patients with patellar fracture on preoperative double lower limbs DUS, especially after the injury, the official guidelines suggest don't regular scans, but the results of this study showed that the preoperative blood clots in the patellar fracture patients have a higher risk, so for fracture patients, not just the patellar fracture patients, should be thoroughly DVT after hospital screening, and surgery as soon as possible.

Reports have shown that D-dimer is a highly sensitive laboratory marker for DVT [29, 30]. Our results showed that D-dimer levels above 0.5 ug/ml at admission were associated with a 47% increased risk of DVT, which was comparable to the findings of Zhang et al.'s study of lower extremity fractures [2]. D-dimer is a fibrin degradation marker that represents secondary fibrinolytic activity in the blood, which has clinical value in the diagnosis of thrombotic events [31, 32]. Yamasaki et al. [33] retrospectively collected 588 patients undergoing lumbar spine surgery and found that the risk of DVT increased by 9% with D-dimer level greater than 19.2 ug/ml one week after surgery. However, the cut-off values used in various research were highly variable, which may be due to the heterogeneity of the subjects and the study designs. Therefore, the age-adjusted D-dimer levels could be a better predictive model clinically. It is important to note that the incidence of preoperative thrombosis was 5.8%(48/827) in all of our patients with patellar fractures, despite thrombosis prophylaxis, thus indirectly highlighting the importance of breakthrough thrombosis.

Our study presents several limitations. Firstly, since it was a retrospective study, some information might have been missed during data collection. We did not capture the data regarding the past history of thrombosis or family history, which is a limitation of this study. Secondly, we might have under-report DVTs since we used DUS, which has a relatively lower sensitivity compared to CT or MRI angiography. Considering that angiography is an invasive examination, routine DUS for DVT screening is acceptable and is generally used in most medical centers. Thirdly, patients in both groups did not perform off-bed weight-bearing exercises preoperatively because of pain related to the fracture, which may affect the occurrence of DVT. Therefore, the causative relationship of variables with DVT could not be established. Instead, there is a combination that should be cautiously during interpretation.

## Conclusions

In summary, when patients were not allowed to get out of bed and exercise before surgery, the incidence of preoperative DVT following an isolated patellar fracture was 5.8%. Age (each increase 1 year), delay from injury to DUS, and elevated plasma D-dimer levels were independent risk factors for preoperative thrombosis. Despite a low prevalence of DVT after an isolated patellar fracture, this study underscores the importance of identifying those at a high risk of developing DVT, especially those with multiple identifiable factors, and encourage the early targeted use of anti-thromboembolic agents to reduce DVT occurrence.

## Data Availability

The datasets generated and/or analysed during the current study are not publicly available due to limitations of ethical approval involving the patient data and anonymity but are available from the corresponding author on reasonable request.
